# Digital Nativity, Computer Self-Efficacy, and Technology Adoption: A Study Among University Faculties in China

**DOI:** 10.3389/fpsyg.2021.746292

**Published:** 2021-09-21

**Authors:** Chen Zhao, Lei Zhao

**Affiliations:** College of Science and Information Science, Qingdao Agricultural University, Qingdao, China

**Keywords:** technology acceptance, digital nativity, computer self-efficacy, university faculties, China

## Abstract

Technology acceptance and usage become obligatory for people when their work modes change as a result of an unexpected but irresistible force. This is especially true for teachers who are reluctant technology adopters compared with their students. During the COVID-19 pandemic, the Chinese government issued national policies to enforce online teaching and learning. As the success of online teaching largely depends on university faculties' readiness and intentions, how they perceive and practice technology adoption becomes an issue that warrants in-depth research. Unlike their students who grow up with technology and can be seen as digital natives, university faculties may lack competence in using technology, whether to teach or do other tasks. Previous studies on faculties' technology adoption were all conducted in situations where they made volitional decisions to use technology, but their mandatory technology use received scant attention. In addition, although studies suggested that teachers demonstrated features of digital natives, it remains unknown whether or to what extent their digital nativity correlates with technology intentions. To address these research gaps, the current study examined Chinese university faculties' intentions to use technology for online teaching by incorporating digital nativity and computer self-efficacy as key determinants into technology acceptance variables. Results suggested that digital nativity was a key factor that affected university faculties' online teaching, as evidenced by the fact that 67% of the variance could be explained by perceived usefulness, attitudes and digital nativity. In addition, it was also found that computer efficacy significantly influenced perceived ease of use.

## Introduction

Digitalization encourages people of different professions to improve their job performance with information and communication technologies. With COVID-19 sweeping the world, technology integration in education has greatly aroused educational stakeholders' attention and discussion. To ensured continued teaching and learning, university faculties resorted to various synchronous online teaching tools such as Zoom, Microsoft Teams, and Tencent Classroom (Huang et al., 2021b). Although this drastic teaching-mode shift facilitates teachers' pedagogical design thinking on technology (Tsai and Chai, 2012), it brings great cognitive, emotional, and technical challenges to those who are not familiar with technology-enhanced teaching. It is, therefore, necessary to examine university faculties' perceptions of technology adoption in teaching.

Ever since Prensky (2001) made the distinction between digital natives and digital immigrants based on thinking and behavior patterns, scholars have conducted research to assess technology users' digital nativity (Çoklar et al., 2017; Huang et al., 2021c). Although it remains arguable whether age is one of the key criteria in defining digital natives (Kennedy et al., 2008), researchers (e.g., Teo, 2013) adopted Prensky (2001)'s definition to develop the Digital Nativity Assessment Scale to assess digital nativity. Building on Prensky's interpretation of features of digital natives, namely, grow up with technology, comfortable with multi-tasking, reliant on graphics for communication and thrive on instant gratification and rewards, existing studies on digital natives and their technology adoption mostly focused on student cohort (e.g., Bennett and Maton, 2010; Teo, 2013; Chen et al., 2016; Tran et al., 2020), with few studies examining adult users' digital nativity. To address this research gap, studies conducted in a higher education context suggested that university teachers also demonstrated features of digital natives (Smith et al., 2020; Huang et al., 2021c), which empirically supported scholars' doubt about defining digital nativity by age. Despite these efforts, it remains unknown whether and to what extent digital nativity is related to university teachers' technology adoption.

As the first country enforcing the COVID-19 pandemic lockdown, China was among the first to move from traditional classroom teaching to full online teaching under the initiative of “Suspending Classes without Stopping Learning” (Huang et al., 2021b). This drastic shift may bring about changes in university faculties' perceptions and present significant challenges for teachers' adaption to online teaching. Therefore, it is necessary to investigate their online teaching during the pandemic lockdown. The research question of the current study is: To what extent the research model explains university faculties' online teaching during the pandemic? To answer the research question, the study used a survey to inquire into university faculties' responses to technology adoption.

## Model Development

### Technology Acceptance Model

Widely used by scholars to unpack users' intentions of technology use internationally, the technology acceptance model (TAM) proposed by Davis (1989) is suggested as one of the most valid theories in predicting users' technology acceptance (King and He, 2006; Al-Emran et al., 2018; Granić and Marangunić, 2019; Huang et al., 2021a,b). In the TAM, there are two main variables that explain users' intentions: Perceived usefulness (PU) and perceived ease of use (PEU) (Davis, 1989). PU refers to one's belief that using technology helps improve work performance and efficiency (Teo, 2009), while PEU indicates a belief about using technologies without any effort. According to Davis (1989), PEU influences PU and both of them are associated with attitudes (ATU) which measures one's fondness of technology. Behavior intention (BI) measures the degree of one's aspiration or willingness to use technology and is influenced by both ATU and PU (Davis, 1989; Teo, 2009). In previous studies contextualized in Chinese higher educational settings, Teo et al. (2018) accepted the TAM as valid in explaining Chinese English teachers' intentions to use technology. Those discussions gave rise to the following hypotheses.

H1: PU is significantly related to ATU.

H2: PU is significantly related to BI.

H3: PEU is significantly related to PU.

H4: PEU is significantly related to ATU.

H5: ATU is significantly related to BI.

### Extended Variables: Computer Self-Efficacy and Digital Natives

An extended variable in this study is computer self-efficacy (CSE), which measures the extent to which an individual believes that he or she has the ability to perform online teaching with technology (Compeau and Higgins, 1995). CSE derives from Bandura (1977)'s notion of self-efficacy that gauges the extent to which one perceives that he or she has the ability to organize, execute actions and achieve specific goals. In technology acceptance studies, researchers found that CSE played a significant role in influencing users' perceived ease of use (Teo et al., 2018; Bai et al., 2021; Huang and Teo, 2021). In this study, we included CSE as an extended variable because we believe individuals with a high level of CSE may not easily feel anxious or frustrated when they face unexpected challenges, such as the emergent shift to online teaching as a result of COVID-19 in particular. In addition, they are more likely to overcome technological difficulties (Compeau and Higgins, 1995) and thus perceive online teaching as a not-too-difficult or even a handy task. In light of this revelation, another hypothesis arises.

H6: CSE is significantly associated with PEU.

Another important variable included in this study is digital nativity (DN) which characterizes new generation's technology preference and behavior. In 2001, Prensky proposed that digital natives were a new generation of learners who entered higher education, and that they were different from digital immigrants because they were naturally digitally literate and inherently proficient users of technology. Prensky refers to digital natives as those who grow up with technology, demonstrate features of parallel processing and multitasking, and usually rely on technology to perform diverse tasks, such as learning and interpersonal communication. Unlike digital immigrants who are not used to using technology to perform tasks, digital natives prefer using graphics to express ideas and expect prompt responses (Prensky, 2001; Teo, 2013). Although Prensky considered people who are born after the 1980s as typical digital natives, scholars argued that digital natives were not an identifiable generation solely defined by age (Bennett and Maton, 2010; Huang et al., 2021c). Many people who did not grow up in a pervasive ICT environment also demonstrate features of digital natives if they become familiar with ICT through constant use of technology (Ransdell et al., 2011). This argument was validated by Huang et al. (2021c)'s study of English teachers in a Chinese higher educational context. To further unpack its influence on faculty members' intentions to adopt online teaching during the pandemic, the current study examined their digital nativity (DN) and its impact on their online teaching intentions by associating DN with the main variables of the TAM (PU, PEU, ATU, and BI) as well as the CSE.

**Figure 1:**
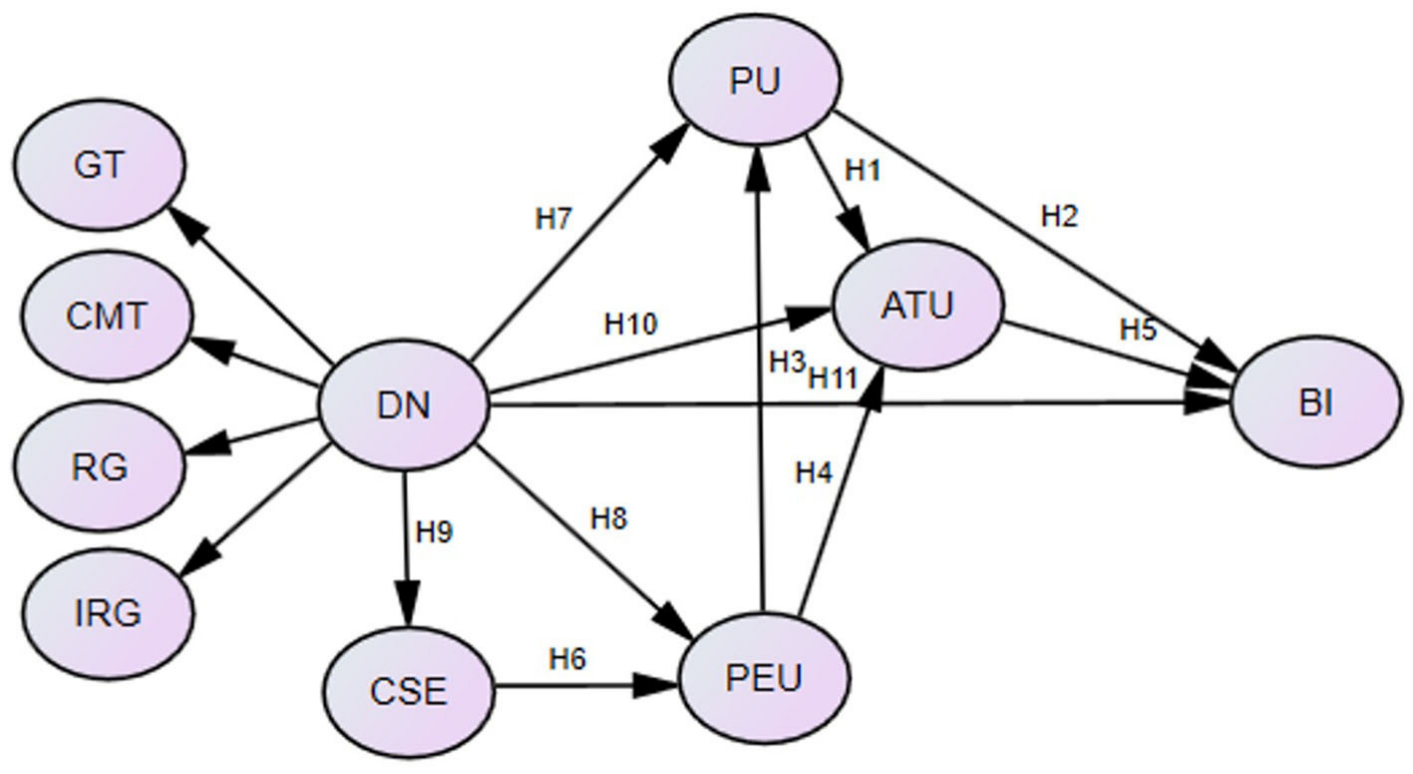
The research model. PU, perceived usefulness; PEU, perceived ease of use; ATU, attitude; BI, behavioral intention; CSE, computer self-efficacy; DN, digital nativity; GT, grow up with technology; CMT, comfortable with multi-tasking; RG, reliant on graphics for communication; IRG, thrive on instant gratification and rewards.

To achieve this goal, we used the Digital Navies Assessment Scale (DNAS) developed by Teo (2013) to measure university faculties' digital nativity, given that the DNAS has been validated among teachers in diverse cultural contexts (e.g., Teo et al., 2016; Huang et al., 2021c). The DNAS consists of four dimensions: grow up with technology (GT), comfortable with multitasking (CMT), reliant on graphics for communication (RG), and thrive on instant gratification and rewards (IRG), thus giving rise to five additional hypotheses whose interactions with other hypotheses form the research model of the current study (as shown Figure 1).

H7: DN is significantly associated with PU.

H8: DN is significantly associated with PEU.

H9: DN is significantly associated with CSE.

H10: DN is significantly associated with ATU.

H11: DN is significantly associated with BI.

## Materials and Methods

### Participants and Data Collection

This study was conducted in the Spring of 2020. Participants are 502 faculties from 30 universities in China. Approached by our contacts in Chinese universities, they were invited to fill in a survey questionnaire designed for the study. Researchers in this study designed the research questionnaire, converted it into a survey using Wenjuanxing, a widely used online survey tool in China, and distributed it with WetChat, a popular social media app in China.

Before the study, participants were informed of the voluntariness of their participation in the study. They each spent about 15 min filling out the questionnaires. After data collection, responses were carefully checked to ensure the completeness. Among the participants, 77.9% were females and 22.1% were males. Their ages ranged from 23 to 59, with the mean age being 37.6 (SD = 6.77). The mean length of their university work experience was 13 years (SD = 7.44), and the mean year of their technology use in teaching was 9.33 (SD = 5.30).

### Instruments

The questionnaire designed for the research consists of two sections. Section One includes questions to obtain the demographic information of the participants, such as their age, gender, and technology use experience. Section Two comprises a series of items underlying constructs adapted from various sources in which the validity and reliability of the constructs were suggested. Those constructs are: Computer self-efficacy (Compeau and Higgins, 1995; Teo et al., 2018); perceived usefulness, perceived ease of use, attitudes, behavioral intention derived from the technology acceptance model (Davis, 1989); and the Digital Nativity Assessment Scale (DNAS, Teo, 2013). The DNAS includes four sub-constructs: grow up with technology, comfortable with multitasking, reliant on graphics for communication, thrive on instant gratification and rewards. All the items were tested by the 7-point Likert Scale from 1 to 7 (1 stands for “Completely Disagree,” 7 stands for “Completely Agree”).

### Data Analysis

To examine the hypothesized relationships, structural equation modeling (SEM) with the maximum likelihood as the method for estimating parameters was conducted using AMOS 24.0. SEM was chosen as the main data analysis technique because it enabled us to see the relationships among the proposed constructs simultaneously. As suggested by Anderson and Gerbing (1988), we performed a two-step analysis (the measurement model and the structural model). To be specific, the confirmatory factor analysis of the measurement model was set to describe the fit between the observed indicators (items) and the underlying constructs in the sample data, while the structural model was tasked with examining the significance of the hypotheses.

## Results

### Descriptive Statistics

As shown by the descriptive statistics for the constructs in Table 1, faculty members generally responded positively to online teaching, with the mean values of the constructs varying from 4.53 to 5.73. In addition, kurtosis and skewness were assessed for univariate normality by applying the criteria of |3| and |8| (Kline, 2011). Skewness and Kurtosis ranged from −0.40 to 3.50 and from −1.04 to 0.155, respectively, indicating that the data demonstrated a normal distribution.

**Table 1:** Information of the constructs.

**Constructs**	***N*** **of items**	**Minimum**	**Maximum**	**Mean**	**SD**
Perceived usefulness	6	1.00	7.00	5.84	0.87
Perceived ease of use	4	1.60	7.00	4.92	0.95
Attitudes	5	1.80	7.00	5.45	0.90
Computer self-efficacy	7	1.86	7.00	5.16	0.75
Behavioral intention	5	2.00	7.00	5.69	0.90
Grow up with technology	3	1.00	7.00	5.73	0.98
Comfortable with multi-tasking	5	1.67	7.00	5.05	1.11
Reliant on graphics for communication	5	1.00	7.00	4.53	1.04
Thrive on instant gratification and rewards	4	2.00	7.00	5.32	0.89

### Testing the Measurement Model

The analysis of the measurement model shows the factor loadings of the underlying constructs and the model fit between the observed indicators and the underlying constructs. The confirmatory factor analysis (CFA) with the use of maximum likelihood estimation was performed. The value of Mardia's coefficient was 638.307, much lower than the recommended value of 2024 which is calculated as *p* (*p* + 2), where *p* indicates the total number of observed items. This suggested the multivariate normality was achieved and thus adequate for the confirmatory factor analysis (Raykov and Marcoulides, 2011). The factor loadings of the constructs are shown in Table 2. Most item loadings were near or above 0.6, indicating the significance of these items to their underlying constructs (Hair et al., 2010). To test the reliability and convergent validity of the constructs, composite reliability (CR) and average variance extraction (AVE) were used based on the acceptable level of 0.70 (Gefen et al., 2000) and 0.50 (Fornell and Larcker, 1981), respectively. Table 2 indicates that CRs and AVEs met the above criteria, except for the AVE of CSE, suggesting that the items used in this study generally possess adequate psychometric properties. Besides, the model fit indices used in this study are the ratio of the minimum fit function to its degree of freedom (χ^2^/df), with a value lower than 3.0 to be considered desirable (Carmines and McIver, 1981); the Comparative Fit Index (CFI) and Tucker-Lewis Index (TLI), with values >0.90 indicating a good fit (Hair et al., 2010); the Root-Mean-Square Error of Approximation (RMSEA) and the Standardized Root Mean Square Residual (SRMR), with values lower than 0.08 indicating a good model fit (Hair et al., 2010). Based on these criteria, results of this study indicated that the measurement model had achieved a good model fit (χ^2^/df = 2.248, TLI = 0.916, CFI = 0.923, SRMR = 0.0579, and RMSEA = 0.050 [0.047, 0.053]).

**Table 2:** Factor loadings of the constructs.

**Factors**	**Indicators**	**Loadings**	**CR**	**AVE**
Perceived usefulness (PU)	PU1	0.778	0.897	0.593
	PU2	0.796		
	PU3	0.71		
	PU4	0.773		
	PU5	0.688		
	PU6	0.864		
Perceived ease of use (PEU)	PEU1	0.717	0.872	0.631
	PEU2	0.785		
	PEU3	0.871		
	PEU4	0.796		
Attitude (ATU)	ATU1	0.822	0.874	0.582
	ATU2	0.792		
	ATU3	0.637		
	ATU4	0.794		
	ATU5	0.756		
Behavioral intention (BI)	BI1	0.799	0.923	0.708
	BI2	0.896		
	BI3	0.926		
	BI4	0.744		
	BI5	0.83		
Grow up with technology (GT)	GT1	0.816	0.879	0.708
	GT2	0.897		
	GT3	0.808		
Comfortable with multitasking (CMT)	CMT1	0.66	0.874	0.583
	CMT2	0.723		
	CMT3	0.816		
	CMT4	0.793		
	CMT5	0.815		
Reliant on graphics for communication (RG)	RG1	0.723	0.874	0.583
	RG2	0.822		
	RG3	0.796		
	RG4	0.828		
	RG5	0.629		
Thrive on instant gratification and rewards (IRG)	IGR1	0.779	0.807	0.513
	IGR2	0.719		
	IGR3	0.746		
	IGR4	0.61		
Computer self-efficacy (CSE)	CSE1	0.596	0.864	0.477
	CSE2	0.721		
	CSE3	0.719		
	CSE4	0.56		
	CSE5	0.75		
	CSE6	0.752		
	CSE7	0.713		

*CR, composite reliability; AVE, average variance extracted*.

### Testing the Structural Model

After ensuring the reliability and the validity of the measurement model, we further tested the structural model to examine hypothesized relationships in the research model, as well as the percentage of the variance predicted. The structural model achieved a good model fit (χ^2^/df = 2.382, CFI = 0.913, TLI = 0.907, RMSEA = 0.053 [0.050, 0.055], SRMR = 0.0728). Table 3 shows the results of the hypotheses proposed in the research model.

**Table 3:** Results of the hypotheses.

**Hypotheses**	**Paths**	**Path coefficients**	**Results**
H1	PU → ATU	0.529^***^	Supported
H2	PU → BI	0.042	Not supported
H3	PEU → PU	0.07	Not supported
H4	PEU → ATU	0.243^***^	Supported
H5	ATU → BI	0.291^***^	Supported
H6	CSE → PEU	0.377^***^	Supported
H7	DN → PU	0.620^***^	Supported
H8	DN → PEU	0.159^*^	Supported
H9	DN → CSE	0.677^***^	Supported
H10	DN → ATU	0.240^***^	Supported
H11	DN → BI	0.558^***^	Supported

*** *p < 0.001;*

* *p < 0.01*.

The proposed research model explained 67% of university faculties' technology adoption intentions. Variances explained for ATU, PEU, CSE, and PU are 69, 25, 46, and 43%, respectively. Besides, all the four sub-constructs measuring digital nativity achieved significance with their path coefficients being 0.767 (grow up with technology, GT), 0.443 (comfortable with multi-tasking, CMT), 0.390 (reliant on graphics for communication, RG) and 0.761 (thrive on instant gratification and rewards, IRG).

As for university faculties' digital nativity, results indicated that they demonstrated features of digital natives with their mean values all above 4 (Table 1). In addition, their digital nativity was significantly associated with perceived usefulness (H7), perceived ease of use (H8), computer self-efficacy (H9), attitudes (H10), and behavioral intentions (H11).

Perceived usefulness and perceived ease of use were both related to faculties' attitudes toward technology use (H1 and H4), which further impacts their behavioral intention (H5). However, unlike the original technology acceptance model (Davis, 1989), the relationships between perceived usefulness and behavioral intentions (H2) and between perceived ease of use and perceived usefulness (H3) were not significant in this study. Based on the results, cultural and contextual reasons were discussed to support the results.

## Discussion

The results of this study evidence the validity of the TAM to predict teachers' intentions to use technology (Teo et al., 2018; Huang and Teo, 2021), as well as the important role digital nativity played in technology adoption of university faculties, which highlights the importance of incorporating constructs like digital nativity to examine users' technology adoption. This is especially rationale since many university faculties are digital immigrants and they need to pick up technology skills to cope with job requirements in the digital age. Overall, the research model is able to explain 67% of BI variance mainly through digital nativity, perceived usefulness and attitudes. Additionally, the research model explains high percentages of the variances of the three core variables from the TAM, namely, 69% of the variance of ATU, 43% of PU, and 25% of PEU.

The hypotheses of the research model are mostly supported. Consistent with the TAM and previous studies, perceived usefulness is related to university faculties' attitudes toward online teaching (Davis, 1989; Huang et al., 2021b; Khlaisanga et al., 2021), which further explains their intentions to adopt online teaching (Sánchez-Prieto et al., 2019). This indicated that when university faculties believe using technology is useful, such as enabling them to conduct teaching and administrative tasks even at the epidemic quarantine, they would form an opposite attitude toward technology use and this feeling directly leads to their technology adoption intentions. Besides perceived usefulness, perceived ease of use is also associated with attitudes, indicating that when university faculties perceived online teaching as a useful and handy task, they would be more likely to be fond of online teaching (Davis, 1989; Teo and Huang, 2019; Khlaisanga et al., 2021). As one of the key factors that examine individuals' self-perceived ability, computer self-efficacy is suggested to relate to university faculties' perceived ease of use, indicating that when they believe they have a sufficient ability to teach online, they would not consider the task as difficult or laborious (Venkatesh and Bala, 2010; Dong et al., 2020). The supported relationship between computer self-efficacy and perceived ease of use is reasonable because self-efficacy measures the degree to which one believes he or she can perform a certain task. For university faculties, when they believe they have sufficient abilities to perform tasks by using technology, such as the emergency remote teaching, they would be very likely to perceive online teaching as an easy task. For those with low computer self-efficacy, online teaching may bring anxiety or confusion to them, and this psychological mindset would impede their intentions to teach online.

As an important annectant proposed in the study, digital nativity is significantly related to PU, PEU, CSE, ATU, and BI, demonstrating its power of predicting university faculties' online teaching adoption. As previous studies suggested (Huang et al., 2019), university teachers demonstrated features of digital natives although many of them were born before the 1980s. However, the presence of such features does not necessarily mean that they have acquired necessary professional skills to fulfill their teaching and administrative tasks, especially with respect to the ability to use technology for such tasks. It is reasonable to believe that when university faculties consider they have grasped necessary technological skills and can act as digital natives, they would think using technology is useful and easy, consider themselves as competent in these skills, form a positive attitude toward technology integration, and ultimately be more likely to engage in technology-enabled teaching.

Inconsistent with the TAM, the relationship between PU and BI did not achieve significance in this study. Since Chinese people often hold collectivist values which strengthen group interests and conformity in thinking and behavior (Hofstede, 2008; Huang et al., 2019), their behaviors are greatly influenced by higher authorities. During the pandemic, Chinese people's collectivism was increasingly strengthened due to the Chinese government's fast and effective response to the pandemic. Therefore, university faculties' decision making may be more likely to be influenced by policy requirement as suggested by Huang and Teo (2021). In addition, the fact that perceived ease of use was not significantly related to perceived usefulness calls for further consideration of antecedents of perceived usefulness by considering cultural and contextual features.

### Limitations and Future Study

This study has some limitations. First, this study used online survey due to the impact of the pandemic. Compared with the traditional paper-and-pencil surveys that provide higher response rates, online survey would not receive a high response rate given the fact that some potential participants are lack of willingness to participate in online survey (Lefever et al., 2007). Second, participants might respond to research questions in a socially desirable direction instead of expressing their true feelings (Richman et al., 1999). Thirdly, during the pandemic, many university faculties were busy with online teaching, compared with the large population in China, the sample size of the study is relatively not big enough and thus, lowered the generalizability of the study results. Therefore, further studies are suggested to diversify data collection methods, increase sample size, involve teachers in diverse subjects to achieve greater generalizability regarding faculties' online teaching. Fourth, the cross-sectional design is also the research limitations and further study would consider diverse research design such as experimental study to unpack causal relationships among the variables. Finally, factors such as age and gender were not examined in the current study and further study is necessary to unpack their influences on technology acceptance.

## Conclusion

Contextualized in Chinese higher institutional context, this study investigated university faculties' digital nativity and its association with their online teaching intentions during the COVID-19 pandemic. Based on the results of the study, university faculties demonstrated features of digital nativity, which is related to the computer self-efficacy and the main factors proposed in the original technology acceptance model. This indicates scholars should revisit the notion of digital natives and include it in considering technology users' intentions to use technology. Results also provide suggestions for both administers of teacher training programs and policy makers to improve teacher professional development, such as the improvement in technical support, and provision of technology training.

## Data Availability Statement

The raw data supporting the conclusions of this article will be made available by the authors, without undue reservation.

## Ethics Statement

Ethical review and approval was not required for the study on human participants in accordance with the institutional requirements. The participants voluntarily participated in this study and provided their informed consent to participate in this study.

## Author's Note

Contextualized in Chinese universities, this study contributed to people's understanding of technology acceptance theories by unpacking faculties' digital nativity and its association with computer self-efficacy as well as their technology adoption during the COVID-19 pandemic. Results provided evidence to support the presence of factors that impact faculties' technology adoption during the pandemic. Taking account of those factors and their influences, policy makers, and university administrators can make informed decisions on technology-related issues accordingly.

## Author Contributions

CZ: conceptualize, data analysis, and writing. LZ: supervision and revision. Both authors contributed to the article and approved the submitted version.

## Funding

This study was supported by the project, Exploring and practicing curriculum reform integrating ideological teaching based on the All Three Education Framework (Grant no.: M2020013).

## Conflict of Interest

The authors declare that the research was conducted in the absence of any commercial or financial relationships that could be construed as a potential conflict of interest.

## Publisher's Note

All claims expressed in this article are solely those of the authors and do not necessarily represent those of their affiliated organizations, or those of the publisher, the editors and the reviewers. Any product that may be evaluated in this article, or claim that may be made by its manufacturer, is not guaranteed or endorsed by the publisher.
